# Differential DAMP release was observed in the sputum of COPD, asthma and asthma-COPD overlap (ACO) patients

**DOI:** 10.1038/s41598-019-55502-2

**Published:** 2019-12-17

**Authors:** Xiaolin Huang, Xiaoyu Tan, Yue Liang, Changchun Hou, Dongming Qu, Mengze Li, Qinghua Huang

**Affiliations:** 1 Department of intensive care unit, The second affiliated hospital of Guangxi Medical University, Nanning, 530021 China; 2 Department of respiratory medicine, The second affiliated hospital of Guangxi Medical University, Nanning, 530021 China; 3 The Eighth People’s Hospital of Nanning City, Nanning, 530021 China; 4 Department of respiratory medicine, The first affiliated hospital of Guangxi Medical University Nanning, Nanning, 530021 China

**Keywords:** Asthma, Chronic obstructive pulmonary disease

## Abstract

Asthma-COPD overlap (ACO) has been under intensive focus; however, the levels of damage-associated molecular patterns (DAMPs) that can activate the innate and adaptive immune responses of ACO are unknown. The present study aimed to examine the levels of some DAMPs in asthma, COPD, and ACO and to identify the associations between clinical characteristics and DAMPs in ACO. Sputum from subjects with asthma (n = 87) or COPD (n = 73) and ACO (n = 68) or from smokers (n = 62) and never-smokers (n = 62) was analyzed for high mobility group protein B1 (HMGB1), heat shock protein 70 (HSP70), LL-37, S100A8, and galectin-3 (Gal-3). The concentration of HMGB1, HSP70, LL-37, and S100A8 proteins in sputum from ACO patients was significantly elevated, whereas that of Gal-3 was reduced, compared to that of smokers and never-smokers. The levels of HMGB1 and Gal-3 proteins in ACO patients were elevated compared to those in asthma patients. The sputum from ACO patients showed an increase in the levels of LL-37 and S100A8 proteins compared to that of asthma patients, whereas the levels decreased compared to those of COPD patients. The concentrations of HMGB1, HSP70, LL-37, and S100A8 proteins in the sputum of 352 participants were negatively correlated, whereas the levels of Gal-3 were positively correlated, with FEV1, FEV1%pred, and FEV1/FVC. Sputum HMGB1 had a high AUC of the ROC curve while distinguishing ACO patients from asthma patients. Meanwhile, sputum LL-37 had a high AUC of the ROC curve in differentiating asthma and COPD. The release of sputum DAMPs in ACO may be involved in chronic airway inflammation in ACO; the sputum HMGB1 level might serve as a valuable biomarker for distinguishing ACO from asthma, and the sputum LL-37 level might be a biomarker for differentiating asthma and COPD.

## Introduction

Chronic obstructive pulmonary disease (COPD) and asthma are the most common chronic lung diseases worldwide, and obstructive airway diseases are characterized by airflow limitation and chronic airway inflammation^1,2^. Asthma and COPD differ from each other in their patterns of inflammation, immunological mechanisms, and the reversibility of their airflow limitations. However, some patients, especially elderly patients, may present several features associated with asthma and COPD, thereby complicating the diagnosis^3^. Recently, these patients have been termed asthma-COPD overlap (ACO) by international guidelines, which define ACO as a persistent airflow limitation along with high airflow reversibility^1,2^. Some studies demonstrated that these ACO patients might possess distinct clinical characteristics, such as cough or frequent and severe exacerbations, compared to patients with COPD or asthma^4^. Recently, great progress has been made in understanding the pathophysiology of asthma and COPD; however, the biological features of ACO are still poorly understood.

Airway inflammation plays an essential role in the development of both asthma and COPD. COPD is associated with an increased inflammatory reaction in the lungs, causing destruction of lung parenchyma. Asthma is associated with local chronic inflammation of the airway, resulting in airway remodeling and airway hyperresponsiveness^1,2^. Chronic airway inflammation in asthma and COPD is characterized by activation of the innate and adaptive immune systems^5^. However, the initiation of innate and adaptive immune responses in COPD and asthma has yet to be elucidated. Molecules derived from proteolytic allergens, such as viruses and bacteria, can activate pattern recognition receptors (PRRs) on structural and innate immune cells of the lung to induce the immune response^5^. Importantly, injured or dying cells release damage-associated molecular patterns (DAMPs) that might alarm the immune system by interacting with PRRs^6,7^. Therefore, Pouwels *et al*. postulated that DAMPs play a vital role in the initiation of chronic airway inflammation in COPD^8^.

DAMPs can be classified based on their origin. DAMPs include several nuclear proteins, such as high mobility group protein B1 (HMGB1), IL-1a, and IL-33, cellular organelles, such as mitochondria and histones, and other cellular components, such as adenosine triphosphate (ATP), uric acid, HSPs, heme, haptoglobin, defensins, LL-37, EDN, galectin-3 (Gal-3), heparan sulfate, hyaluronan, and S100 proteins^6,7^.

Increased levels of several DAMPs, including HMGB1, HSPs, and S100A8, have been observed in induced sputum and serum of asthma and COPD patients in our and other previous studies^9–11^ that are implicated in the pathogenesis of asthma and COPD. Thus, HMGB1, HSPs, S100 proteins, and IL-33 have been under intensive focus in addition to other DAMPs, such as EDN, defensin, heme, and IL-1a; in particular, HMGB1 is widely considered the most classic DAMP^6^. Interestingly, accumulating evidence strongly supports the role of Gal-3 in the activation of allergic inflammation, such as eosinophil recruitment, airway remodeling, and development of a Th2 phenotype^12,13^. Similarly, some studies have shown that LL-37 is linked to airway inflammation in asthma and COPD^14^. To deduce the pathophysiology of ACO, we selected a panel of five DAMPs, including HMGB1, HSP70, LL-37, S100A8, and Gal-3, in the induced sputum.

In this study, the levels of five DAMPs in the sputum of patients with asthma, COPD, and ACO were examined, followed by an analysis of the correlations between the concentrations of these DAMPs and different clinical variables.

## Materials and Methods

### Participants

The induced sputum samples were collected from 352 participants (232 patients from the Department of Respiratory Medicine of the Second Affiliated Hospital of Guangxi Medical University and 120 patients from the Eighth People’s Hospital of Nanning City). The participants were divided into five groups according to their medical history and pulmonary function tests: healthy never-smokers (NS, n = 62), healthy smokers (HS, n = 62), asthma patients (asthma, n = 87), COPD patients (COPD, n = 73), and ACO patients (as defined below, n = 68).

The 62 healthy never-smokers were excluded if they had respiratory diseases, such as asthma, COPD, pneumonia, or failed to provide sputum samples. Additionally, all healthy smokers were excluded if they had respiratory diseases. The asthmatic patients were initially diagnosed in the two hospitals, as mentioned above, according to the Global Initiative for Asthma (GINA) guidelines^1^. The exclusion criteria included a respiratory tract infection characterized by purulent sputum and infiltration based on lung X-ray within the previous 1 month. Patients with stable COPD were diagnosed according to the Global Initiative for Chronic Obstructive Lung Disease (GOLD) guidelines with incomplete reversible airflow obstruction. The exclusion criteria for all patients included respiratory tract infection, oral steroid treatment, or an exacerbation during the month before admission and a history of other lung diseases.

The ACO patients were defined as smoking asthmatics with a smoking history ≥20 pack-years and COPD patients younger than an age of 40 years with a history of physician-diagnosed asthma or with reversible airway obstruction.

The present study was approved by the Ethics Committee of Guangxi Medical University according to the 1964 Declaration of Helsinki and its later amendments, and all experiments on humans were performed in accordance with the relevant guidelines. All subjects provided informed consent for participation.

### Pulmonary function tests

Pulmonary function tests before sputum induction were performed using the Jaeger Masterscope spirometry system. Two reproducible measurements of each FEV1, FEV1/FVC, and FEV1%pred were obtained before and after inhalation of 400 mg salbutamol.

### Scale

#### Asthma patients’ symptom score

Asthma and ACO patients were instructed to record their daytime and nocturnal symptoms for the preceding 24 h, as described previously^9^.

#### Medical research council (MRC) scale

COPD and ACO patients were required to record points by reading the 5-point Medical Research Council (MRC) dyspnea scale, as described previously^9^.

### Sputum induction

Sputum induction and processing has been described previously^9^. The sputum induced by inhalation of hypertonic saline was weighed and diluted 4X in 0.1% dithiothreitol (Sigma–Aldrich, St. Louis, MO, USA) in water. This suspension was mixed and incubated in a shaking water bath at 37 °C, followed by centrifugation at 750 × *g* for 10 min. The supernatant was stored at −80 °C, and the slides were stained with hematoxylin-eosin (H&E; Biyuntian, China) for differential cell enumeration (n = 200 cells were counted).

### Measurement of HMGB1, HSP70, LL-37, S100A8, and Gal-3 in sputum supernatants

The HMGB1, HSP70, LL-37, S100A8, and Gal-3 levels were quantified by ELISA kits [HMGB1 (Cat. No. HHE3533), HSP70 (Cat. No. HHE7116, HyperHeal, Shanghai, China); LL-37 (Cat. No. HEK321; Hycult Biotech Inc., German); S100A8 (Cat. No. DY4570-05), and Gal-3 (Cat. No. SGAL30; R&D Systems, Minneapolis, MN, USA] according to the manufacturer’s instructions. The detection limits of HMGB1, HSP70, LL-37, S100A8, and Gal-3 were 0.03 ng/mL, 0.05 ng/mL, 0.01 ng/mL, 31.2 pg/mL, and 0.085 ng/mL, respectively.

### Statistical analyses

The data were analyzed using SPSS 13.0 software and expressed as the mean ± SEM. The comparisons between groups were investigated using ANOVA, followed by the least significant difference (LSD) post hoc test. The frequency data (for example, sex) were analyzed by the χ^2^ test. The correlations between the levels of DAMPs and pulmonary function parameters and sputum cell counts were evaluated by Spearman’s rank test. The receiver operating characteristic (ROC) curves of the DAMPs were drawn to assess the predictive capability for distinguishing ACO patients from those in other groups. Multiple stepwise regression analysis was performed to obtain the predictors of the DAMPs. P-values < 0.05 were considered statistically significant.

## Results

### Clinical characteristics

The clinical characteristics of all participants are shown in Table 1. The five groups had similar sex ratios, body mass indexes (BMIs), and percentages of lymphocytes in the induced sputum. The asthmatics were younger than the participants in the HS, COPD, and ACO groups. Subjects with COPD and ACO had higher counts of sputum cells and percentages of neutrophils and significantly lower lung function compared to NS, HS, and asthma patients. The participants in the HS and ACO groups exhibited greater smoking pack-years than those in the NS and asthma groups but lower smoking pack-years than those with COPD. Patients with ACO had a higher percentage of eosinophils compared to those in the other groups. The percentages of ICS, LABA, and SABA were higher in the COPD and ACO groups than in the other groups.

**Table 1:** The clinical characteristics of all participants.

Characteristic	Healthy never-smokers	Healthy smokers	Asthma	COPD	ACO	P value
n	62	62	87	73	68	
Sex (M/F)	32/30	45/17	48/39	45/28	40/28	0.193
Age (years)	54.23 ± 1.74	57.87 ± 1.57	51.40 ± 1.65*^#^	68.70 ± 1.15*	67.13 ± 0.98	0.002
BMI	23.10 ± 0.80	23.03 ± 0.78	22.08 ± 0.46	20.96 ± 0.50	21.61 ± 0.45	0.065
Never/current/ex-smokers	0/0/0	0/62/0	59/9/19	20/28/25	11/34/23	
Smoking pack-years	0 ± 0^&^	29.39 ± 2.05*	7.47 ± 1.65^&#^	46.86 ± 0.50^&^*^#^	26.59 ± 2.73*	≤0.001
Family asthma history (Y/N)	5/57	3/68	26/61	4/69	19/49	≤0.001
Allergic rhinitis (Y/N)	5/57	4/48	26/61	6/67	17/51	≤0.001
ICS use (Y/N)	0/62	0/62	0/87	44/29	43/25	≤0.001
Short-acting β agonist (Y/N)	0/62	0/62	0/87	28/45	29/39	≤0.001
Long-acting β2 agonist (Y/N)	0/62	0/62	0/87	42/31	39/29	≤0.001
**Post bronchodilator**						
FEV1 (L)	3.24 ± 0.13	3.09 ± 0.11	2.16 ± 0.11*^#&^	1.18 ± 0.41*^#^	1.34 ± 0.039*^#^	≤0.001
FEV1 pred (%)	99.72 ± 2.01	92.69 ± 1.90	74.28 ± 2.99*^#&^	36.74 ± 2.08*^#^	41.08 ± 2.00*^#^	≤0.001
FEV1/FVC (%)	90.18 ± 0.97	86.78 ± 0.98	78.08 ± 1.54*^#&^	50.10 ± 2.06*^#&^	57.68 ± 1.73*^#^	≤0.001
∆FEV1 (%)	3.34 ± 0.47	2.86 ± 0.62	15.37 ± 0.56*^#^	6.62 ± 0.47*^#&^	15.97 ± 0.86*^#^	≤0.001
Daily score			5.96 ± 0.36^&^		7.70 ± 0.32	0.001
Nighttime score			1.03 ± 0.14^&^		1.60 ± 0.16	0.002
MRC dyspnea scale				3.19 ± 0.19	2.87 ± 0.18	0.220
**Sputum cell counts**						
TCC (10^6^/ml)	3.76 ± 0.20	4.14 ± 0.21	4.25 ± 0.18^&^	7.79 ± 0.41*^#^	7.21 ± 0.37*^#^	≤0.001
Macrophages (%)	68.19 ± 2.10	52.32 ± 2.13	46.98 ± 2.38*^&^	28.18 ± 1.51*^#^	22.57 ± 1.60*^#^	≤0.001
Neutrophils (%)	37.90 ± 2.03	43.94 ± 2.04	41.32 ± 2.39^&^	68.06 ± 1.51*^#^	64.97 ± 1.50*^#^	≤0.001
Lymphocytes (%)	2.87 ± 0.50	2.79 ± 0.50	4.53 ± 0.61*^#&^	2.86 ± 0.37*^#^	2.84 ± 0.37*^#^	0.052
Eosinophils (%)	1.10 ± 0.25	0.97 ± 0.21	7.18 ± 0.94*^#&^	0.89 ± 0.11^&^	8.91 ± 0.53*^#^	≤0.001

Data are the means ± SEM. TCC, total cell count; FEV1, forced expiratory volume in 1 s (FEV1); FVC, forced vital capacity. *P < 0.05 versus health never-smokers; ^#^P < 0.05 versus smokers; ^&^P < 0.05 versus ACO.

### Sputum HMGB1, HSP70, and S100A8 levels increased in the asthma, COPD, and ACO groups compared to the HS and NS groups

**Figure 1:**
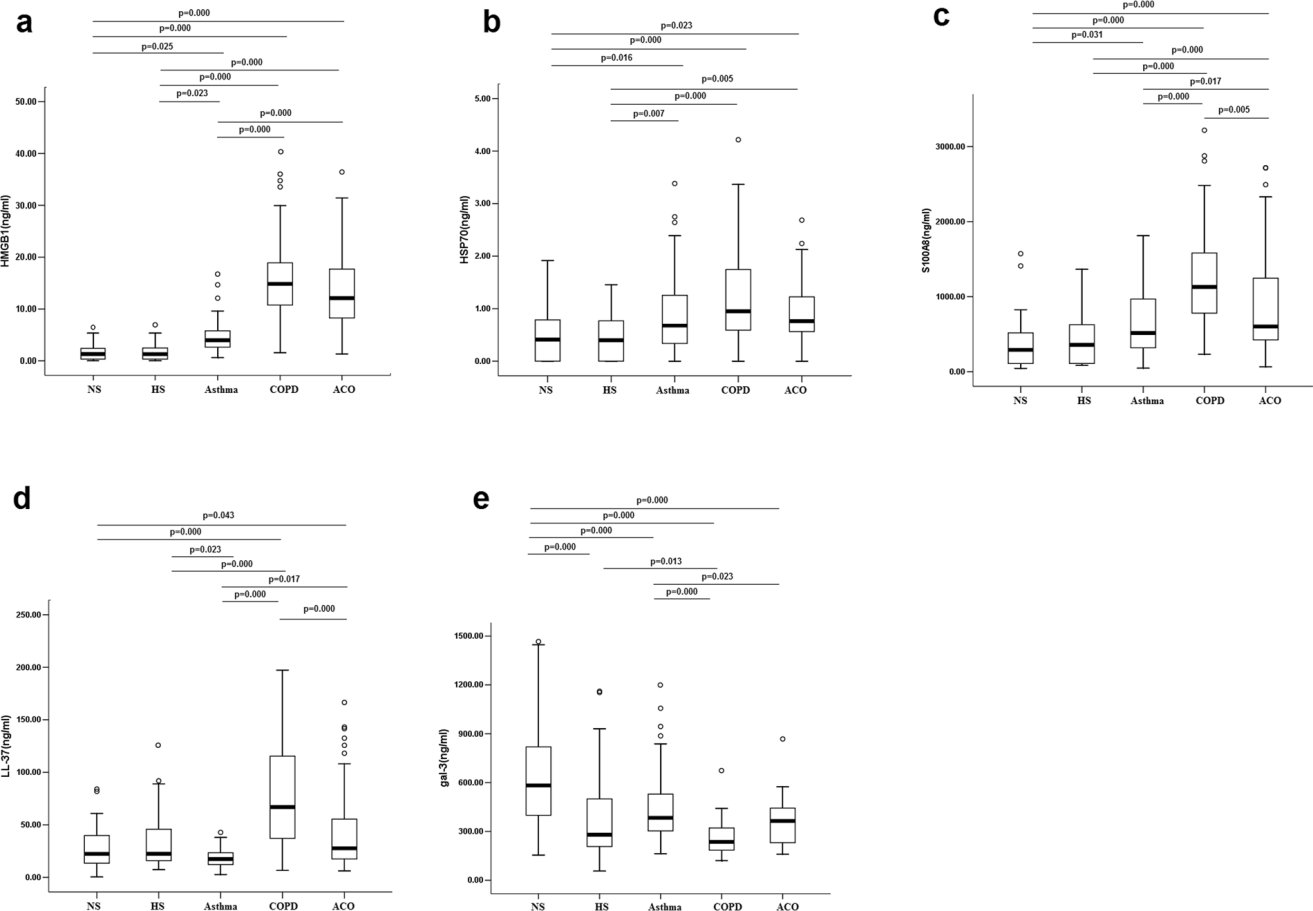
The levels of sputum DAMPs in NS, HS, asthma, COPD, and ACO patients. The lines in the boxes represent the median values, the boxes represent 25–75%, and the lines out of the boxes indicate 10% and 90%, respectively. HS: healthy smoker; NS: never-smoker. ACO: asthma-COPD overlap.

Dithiothreitol (DTT) is routinely selected to dissolve mucus proteins to obtain sputum supernatant. Although DTT has an effect on the analysis of some inflammatory markers in sputum supernatants such as IL-6, it had no effect on the assay standards for IL-1β or TNF-α^15^. Two standard curves of each ELISA with normal reagent or 0.1% DTT were constructed to establish that DTT did not affect the estimations by ELISA in the present study (see Supplementary Table S1). Compared to NS and HS (1.62 ± 0.27 and 1.59 ± 0.27 ng/mL, respectively; Fig. 1a), the HMGB1 levels in the induced sputum were significantly higher in patients with asthma, COPD, and ACO (4.51 ± 0.39, 15.77 ± 1.32, and 13.59 ± 1.22 ng/mL, respectively). Compared to asthmatics, patients with COPD and ACO had a significantly higher level of HMGB1 (both P ≤ 0.001).

The sputum levels of HSP70 were elevated in the asthma, COPD, and ACO groups (0.89 ± 0.09, 1.14 ± 0.14, and 0.93 ± 0.10 ng/mL, respectively) compared to the NS and HS groups (0.53 ± 0.09 and 0.46 ± 0.07 ng/mL, respectively; Fig. 1b). However, no significant differences were observed in sputum HSP70 levels between the ACO and asthma groups or ACO and COPD groups.

Similarly, S100A8 levels in the asthma, COPD, and ACO groups (647.70 ± 56.68, 1241.93 ± 107.39, and 910.76 ± 104.17 ng/mL, respectively; Fig. 1c) were significantly elevated compared to the NS (385.68 ± 63.18 ng/mL) and HS groups (456.25 ± 62.68 ng/mL). The patients with ACO had greater S100A8 levels than asthmatics (P = 0.017) but had lower S100A8 levels than patients with COPD (P = 0.005).

### Sputum LL-37 increases in COPD and ACO

The sputum levels of LL-37 were elevated in the COPD and ACO groups (78.16 ± 7.77 and 46.03 ± 6.16 ng/mL, respectively) but decreased in the asthma group compared to the HS group (29.82 ± 3.09 ng/mL; Fig. 1d); however, no difference was detected in the LL-37 levels between the asthma and NS groups (16.48 ± 1.18 and 18.74 ± 1.22 ng/mL, respectively). The sputum levels of LL-37 in the COPD group were significantly higher than in the ACO (P ≤ 0.001) and asthma groups (P ≤ 0.001).

### Sputum Gal-3 decreases in asthma, COPD, and ACO compared to NS

Compared to the NS group (631.06 ± 54.23 ng/mL; Fig. 1e), the Gal-3 levels in induced sputum were significantly decreased in the HS, asthma, COPD, and ACO groups (390.65 ± 49.78, 449.83 ± 28.62, 266.31 ± 17.08, and 352.17 ± 20.37 ng/mL, respectively). Additionally, sputum LL-37 levels were decreased significantly in the COPD group compared to the HS (P = 0.013) and asthma (P ≤ 0.001) groups but increased in the asthma group compared to the ACO group (P = 0.023).

### Associations between the levels of sputum DAMPs and clinical characteristics

**Figure 2:**
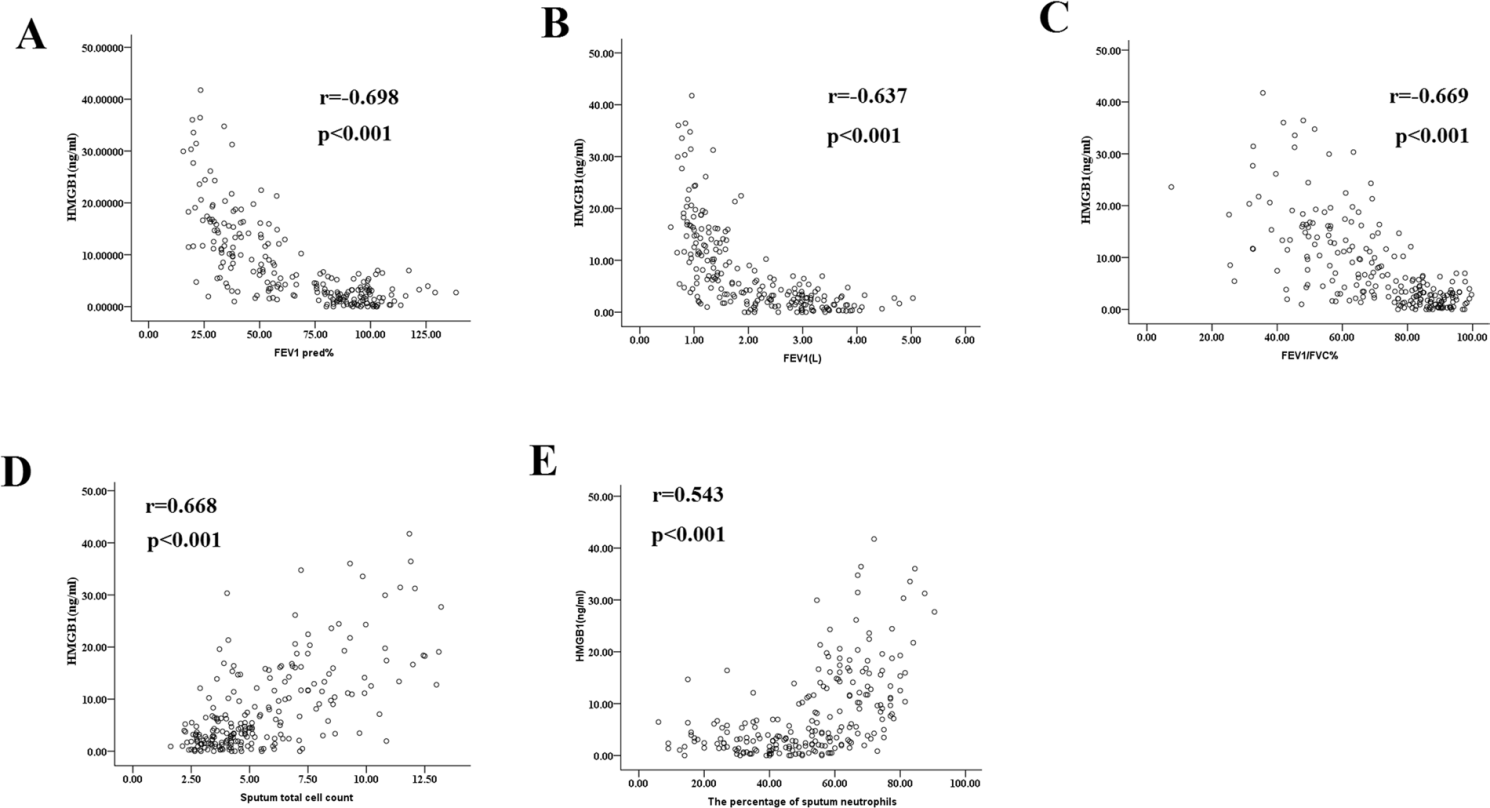
Correlations of the concentrations of HMGB1 in induced sputum with some lung function parameters ((**A**) FEV 1% predicted; (**B**) FEV1; (**C**) FEV1/FVC), sputum total cell count (**D**) and the percentage of sputum neutrophils (**E**) in all study subjects. Correlation coefficients were obtained by the Spearman rank method. FEV1: forced expiratory volume in 1 s; FVC: forced vital capacity.

HMGB1, S100A8, and LL-37 levels in induced sputum in all participants showed a significant negative correlation with some lung function parameters, such as FEV1, FEV1%pred, FEV1/FVC%, and smoking pack-years but a positive correlation with sputum counts and percentage of neutrophils. Conversely, sputum Gal-3 levels in all participants showed a significant positive correlation with FEV1, FEV1%pred, FEV1/FVC%, percentage of macrophages, and smoking pack-years but a negative correlation with sputum counts and percentage of neutrophils (Table 2; Fig. 2). Compared to the other DAMPs, sputum HMGB1 exhibited a robust correlation with the pulmonary function index (Table 2; Fig. 2).

**Table 2:** Correlations between DAMP concentration and clinical characteristics in all study subjects.

Characteristic	HSP70	Gal-3	S100A8	LL-37	HMGB1
BMI	0.001	−0.009	−0.080	−0.064	−0.179*
Smoking pack-years	0.188	−0.277**	0.181*	0.295**	0.343**
Age	0.004	−0.073	0.097	0.208**	0.124
FEV1 (L)	−0.169*	0.279**	−0.687**	−0.328**	−0.637**
FEV1 pred (%)	−0.173	0.278**	−0.685**	−0.376**	−0.698**
FEV1/FVC (%)	−0.173	0.312**	−0.468**	−0.366**	−0.669**
∆FEV1 (%)	0.123	−0.066	−0.057	−0.176*	0.096
TCC (10^6^/ml)	0.101	−0.276**	0.730**	0.377**	0.668**
Macrophages (%)	−0.180*	0.237**	−0.366**	−0.291**	−0.566**
Neutrophils (%)	0.194**	−0.227**	0.385**	−0.357**	0.543**
Lymphocytes (%)	0.003	−0.041	−0.030	−0.164*	0.096

Study participants (N = 352). Data are Spearman rank correlation coefficients between variables. BMI: body mass index; FEV1: forced expiratory volume in 1 s; FVC: forced vital capacity;*p < 0.05; **p < 0.01.

**Figure 3:**
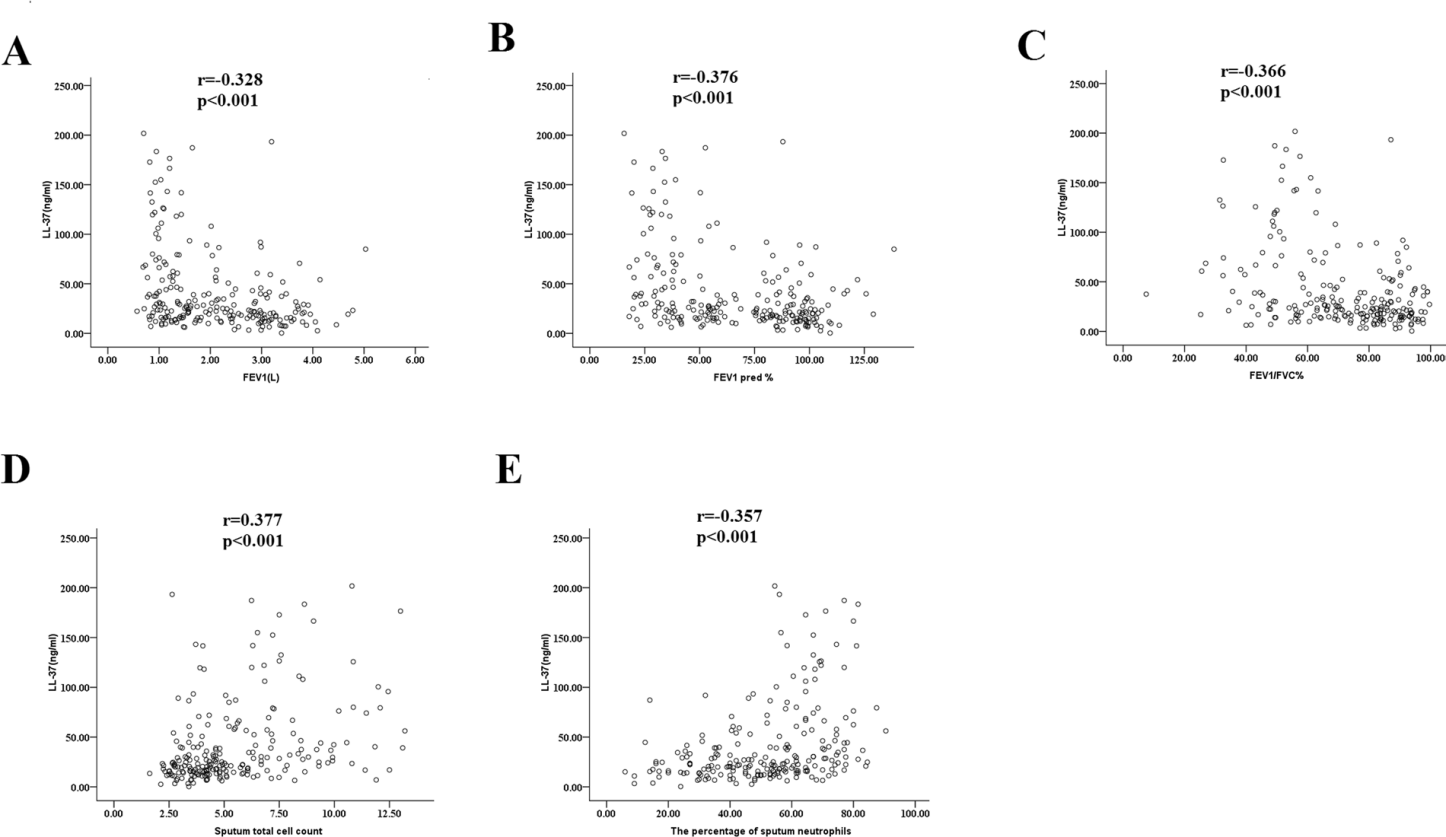
Correlations of the concentrations of LL-37 in induced sputum with some lung function parameters ((**A**) FEV 1% predicted; (**B**) FEV1; (**C**) FEV1/FVC), sputum total cell count (**D**) and the percentage of sputum neutrophils (**E**) in all study subjects. Correlation coefficients were obtained by the Spearman rank method. FEV1: forced expiratory volume in 1 s; FVC: forced vital capacity.

In the ACO group, sputum HMGB1, S100A8, and LL-37 levels correlated negatively with FEV1, FEV1%pred, the percentage of macrophages, and smoking pack-years but positively with the percentage of neutrophils (Table 3; Fig. 3).

**Table 3:** Correlations between DAMP concentrations and clinical characteristics in patients with ACO.

Characteristic	HSP70	Gal-3	S100A8	LL-37	HMGB1
MRC score	−0.101*	−0.185	0.219	0.364	0.311*
FEV1 (L)	−0.049	0.115	−0.522**	−0.273*	−0.464**
FEV1 pred (%)	−0.035	0.133	−0.543**	−0.383**	−0.509**
FEV1/FVC (%)	−0.057	0.124	−0.240	−0.266	−0.366*
**Sputum cell counts**					
TCC (10^6^/ml)	0.031	−0.002	0.314*	0.126	0.377*
Macrophages (%)	0.064	−0.043	−0.327*	−0.337*	−0.394**
Neutrophils (%)	0.104*	0.102	0.338*	−0.408**	0.432**
Lymphocytes (%)	0.143	−0.112	0.098	0.066	0.335*

Study participants (N = 68). Data are Spearman rank correlation coefficients between variables. MRC: Medical Research Council Scale; FEV1: forced expiratory volume in 1 s; FVC: forced vital capacity; *p < 0.05; **p < 0.01.

Multivariate analysis revealed the percentage of neutrophils as the independent predictor for the concentrations of HSP70 and LL-37 in sputum. On the other hand, S100A8 levels were independently correlated with sputum neutrophil counts and FEV1. In addition, FEV1%pred was independently associated with the levels of HMGB1 and LL-37 (Table 4).

**Table 4:** Multivariate analysis was adjusted for sex, age, BMI, smoking pack-years, ICS use and all the significant variables in the univariate analysis. TCC, total cell count.

		B	t	P value
HSP70	Percentage of neutrophils	0.008	2.938	0.004
Gal-3	FEV1/FVC%	3.171	3.515	0.001
S100A8	Sputum neutrophils counts	100.598	5.368	≤0.001
	FEV1	−182.232	−4.336	≤0.001
LL-37	FEV1%pred	−0.597	−6.928	≤0.001
	Percentage of neutrophils	−1.805	−3.884	≤0.001
HMGB1	FEV1%pred	−0.146	−9.265	≤0.001
	TCC	1.100	6.263	≤0.001

Dependent variable: HSP70. R^2^:0.063, adjusted R^2^ :0.068, p = 0.004. Dependent variable: gal-3; R^2^:0.124, adjusted R^2^ :0.116, p = 0.013. Dependent variable: S100A8. R^2^:0.373, adjusted R^2^ :0.346, p ≤ 0.001. Dependent variable: LL-37. R^2^:0.215, adjusted R^2^ :0.204, p = 0.025. Dependent variable: HMGB1. R^2^:0.689, adjusted R^2^ :0.568, p ≤ 0.001.

### Sputum DAMPs differentiate ACO patients from those in other groups

**Figure 4:**
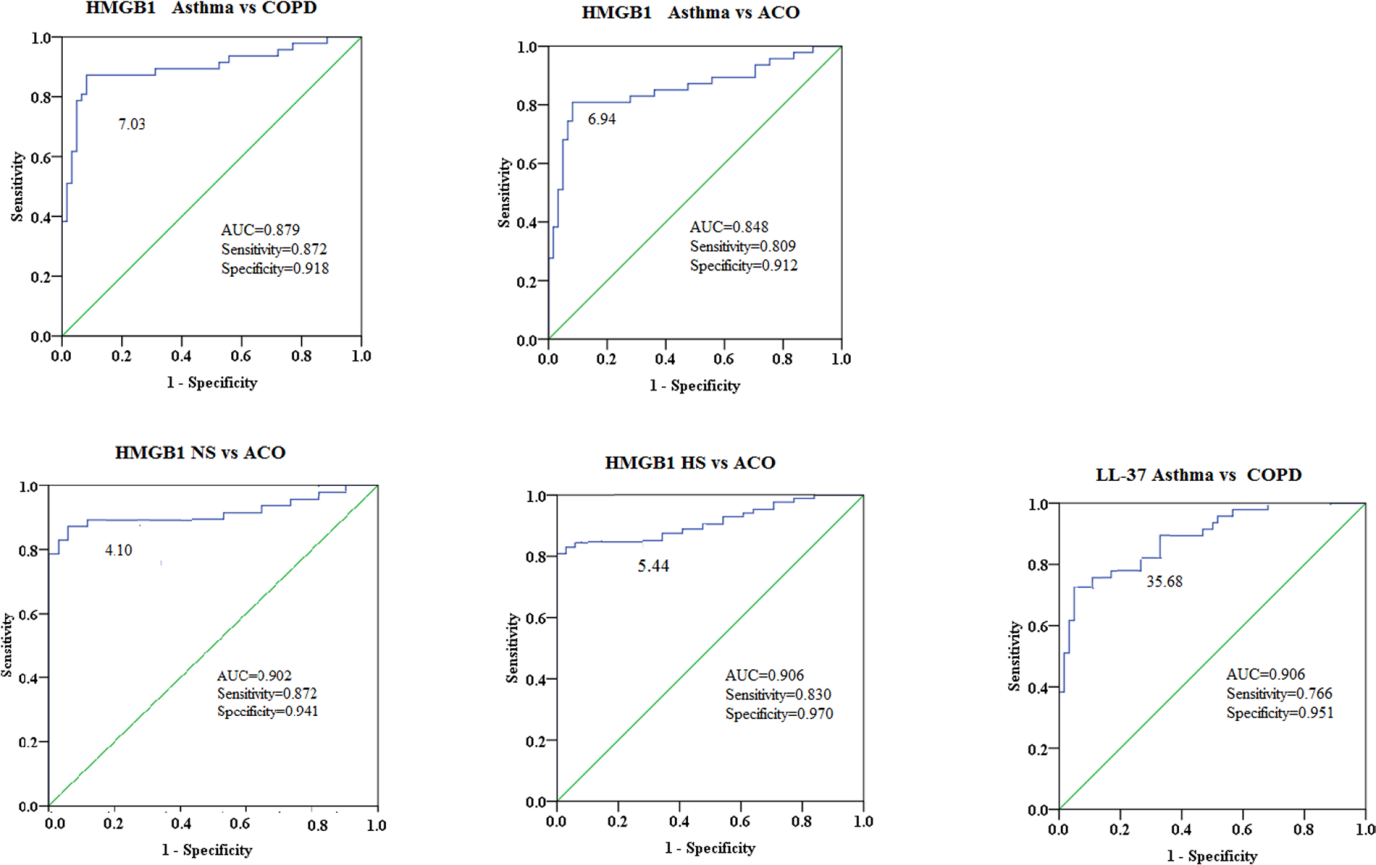
Receiver-operating characteristic analysis of HMGB1 and LL-37 in sputum. NS: health never-smoker; HS: health smoker; ACO: asthma-COPD overlap; AUC: area under the receiver operating characteristic curve.

The accuracy of sputum HMGB1 and LL-37 was analyzed using ROC curves that also differentiated ACO patients from those in other groups (Fig. 4). Sputum HMGB1 displayed a high AUC of the ROC curve that distinguished ACO patients from NS, HS, and asthma patients. Furthermore, sputum LL-37 may be considered a valuable biomarker for differentiating asthma and COPD.

## Discussion

Recently, ACO has been under intensive research. Although several studies have focused on the symptoms and clinical characteristics of ACO, the pathophysiology of this disease has been poorly investigated. In the present study, the levels of sputum HMGB1, HSP70, S100A8, and LL-37 were elevated, while those of sputum Gal-3 were decreased, in patients with ACO compared to healthy controls. Interestingly, the distinct release of these DAMPs between ACO and asthma or COPD indicated that the specific pathophysiology of ACO might differ from that of asthma and COPD. Moreover, sputum HMGB1 may serve as a biomarker for the differentiation of ACO patients from NS, HS, and asthma patients, while sputum LL-37 may differentiate asthmatics from those with COPD. Finally, consistent with previous studies^8,9,11^, we found that the levels of these DAMP molecules in the asthma, COPD and ACO patients were significantly different from those in the normal control groups in our study, indicating the important role of DAMPs in the pathogenesis of COPD.

Although an accurate prevalence of ACO was not detected, 13–73% of all COPD patients and 2% of severe asthmatics fulfilled the criteria for ACO^11,16^. In this study, 29.8% of patients with ACO were similar to that demonstrated previously^16^. In addition, the study^17^ demonstrated that the patients with ACO had a lower lung function parameter, while those in our study had lower smoking pack-years and higher FEV1 and FEV1/FVC% compared to the COPD patients.

The role of DAMPs in the pathophysiology of COPD has been reviewed and discussed by Pouwels *et al*.^8^. Reportedly, HMGB1 levels were elevated in patients with asthma and COPD and independently correlated with the pulmonary function parameters^9^. In a previous study, we also demonstrated that treatment with anti-HMGB1 antibody significantly reversed the development of airway remodeling in a murine asthma model^18^. The elevated levels of HMGB1 in ACO suggested its role in the pathogenesis of ACO as well as asthma and COPD. Moreover, the interaction of HMGB1 with TLR-2, -4, -9, and RAGE can promote the activation of neutrophils, macrophages, and dendritic cells, followed by the release of inflammation mediators^19^. In addition, the sputum HMGB1 levels in the ACO group were negatively correlated with the FEV1%, indicating that HMGB1 in induced sputum may be a useful biomarker for the disease severity of ACO. Strikingly, the current study stated that sputum HMGB1 had a high sensitivity and specificity in distinguishing ACO from NS, HS, and asthma patients and in distinguishing asthmatics from COPD. The sputum HMGB1 has been recognized as a marker reflecting airway neutrophilic inflammation^9,20^. Consistent with these studies, sputum HMGB1 levels were positively correlated with sputum neutrophils in ACO patients. In agreement with the study by Iwamoto *et al*.^21^, the present results suggested that enhanced airway neutrophilic inflammation may be a characteristic feature of ACO.

HSPs are a family of highly conserved proteins in all cells as well as chaperone proteins. Some studies have found that sputum and plasma HSP70 levels in asthmatics were increased and correlated significantly with clinical parameters, such as FEV1 and FEV1/FVC^10,22^. Another study reported that serum HSP70 levels are upregulated in COPD patients compared to healthy controls^23^. In agreement with these studies, the current study revealed that patients with asthma, COPD, and ACO had higher sputum HSP70 levels than healthy never-smokers and smokers. Additionally, neither the role of HSP70 in asthma and COPD nor the mechanism of HSP70 underlying the pathogenesis of ACO has been fully elucidated.

S100A8 has been recognized as a DAMP molecule as extracellular S100 proteins can bind to receptors, such as RAGE and TLR4, and both lead to NF-κB activation^24^. Recently, some studies explored the role of S100A8 in the pathogenesis of asthma and COPD. Mass spectrometry (MS) indicated that S100A8 and S100A9 were elevated in the BALF of COPD patients compared to healthy smokers and never-smokers^11^. Furthermore, a recent computationally intensive analysis of induced sputum proteome demonstrated reduced levels of S100A8/9 in the induced sputum of asthmatic patients compared to healthy subjects^25^. To the best of our knowledge, this is the first report comparing sputum S100A8 in patients with asthma, COPD, and ACO. In the present study, the ACO group showed a higher sputum S100A8 level than asthmatic patients but a lower S100A8 level than COPD patients. Since S100A8 is constitutively expressed in neutrophils, the distinct sputum neutrophil counts may partially account for these differences in the levels of S100A8. However, the association between S100A8 and airway inflammation in these diseases remains unclear.

LL-37/hCAP-18 (one of the antimicrobial peptides), primarily secreted by airway epithelial cells, possesses antimicrobial activity against bacteria, fungi, and viruses^26^. LL-37 also displays a DAMP function as it can provoke a proinflammatory response by binding to TLR7, TLR9, and RAGE and induce necrosis in airway epithelial cells^27^. Consistent with the present study, some studies revealed that the increased LL-37 concentrations in COPD patients negatively correlated with lung function parameters^28,29^. Importantly, ROC curve analysis showed that sputum LL-37 had a high specificity and sensitivity and thus may be a useful and novel biomarker for the differentiation of asthma and COPD. Furthermore, we demonstrated that the sputum LL-37 levels in ACO were significantly higher compared to the non-smoking controls. Based on the previous literature and the current results, we speculated that LL-37 might participate in the inflammatory process underlying ACO.

Gal-3 is a β-galactoside-binding lectin with several physiological functions. Gal-3 is released into the extracellular space and can display a proinflammatory function, termed DAMP^30^. Only a few studies have investigated the role of Gal-3 in asthma and COPD. Gal-3 levels were significantly decreased in the BALF of patients with COPD and healthy smokers compared to controls^31^. Gao *et al*.^32^, for the first time, showed that the concentration of Gal-3 in sputum was significantly reduced in neutrophilic asthma compared to eosinophilic and paucigranulocytic asthma. However, the differences in levels of Gal-3 between asthmatics and healthy controls have not yet been determined. To the best of our knowledge, this is the first report to detect the sputum Gal-3 levels in COPD and ACO patients and to compare the level of Gal-3 in patients with asthma, COPD, and ACO. Interestingly, some studies^31,33^ showed that Gal-3 could remove apoptotic neutrophils, avoiding the amplification of inflammation, and that the decreased level of Gal-3 could be associated with defective efferocytosis of macrophages in COPD.

Notably, the relatively large sample size in the present study was beneficial for minimizing the statistical error. The asthmatics were first diagnosed without the use of medicine; the patients with asthma, COPD, and ACO neither suffered from nor had exacerbations during the month before admission, and this excluded the impact of infection. Furthermore, the participants were selected cautiously, especially the ACO patients, who were diagnosed according to the guidelines; the diagnosis criteria of ACO were based on both clinical history and objective parameters. Nonetheless, the present study has several limitations. Demographic differences were noted between the five groups with respect to age, smoking status, and use of drugs. In addition, comparisons between our results and those of other studies might be challenging due to the lack of consensus on the diagnostic criteria for ACO.

In conclusion, the current data provide a novel approach to understanding the pathophysiological features of ACO. Interestingly, compared to asthma or COPD, ACO exhibited a distinct biomarker profile in sputum DAMP levels. Importantly, sputum HMGB1 is a novel biomarker for differentiating ACO from healthy controls, and sputum LL-37 may also serve as a valuable biomarker for differentiating asthma and COPD.

## Supplementary information

Supplementary information

## Data Availability

The data used to support the findings of this study are included within the article.
