# Supplementary material for: Differential DAMP release was observed in the sputum of COPD, asthma and asthma-COPD overlap (ACO) patients
Source: Sci Rep. 2019 Dec 17;9:19241. doi: 10.1038/s41598-019-55502-2 (PMC6917785; doi:10.1038/s41598-019-55502-2)
Supplement: Supplementary file 1 — Supplementary information [file 41598_2019_55502_MOESM1_ESM.doc]

Supplementary Information

Title: Differential DAMP release was observed in the sputum of COPD, asthma and asthma-COPD overlap (ACO) patients

Authors:

Xiaolin Huang*, Xiaoyu Tan*, Yue Liang, Changchun Hou, Dongming Qu, Mengze Li ,Qinghua Huang

*Xiaolin Huang and Xiaoyu Tan contributed equally to this work.

Supplementary table:

Table 1 The data of two standard curves for each ELISA with normal reagent or 0.1% DTT .

| Concentration of gal-3 (ng/ml) | O.D  Gal-3 without DTT | O.D  Gal-3 with  DTT | Concentration of LL-37(ng/ml) | O.D  LL-37 without DTT | O.D  LL-37 with  DTT | Concentration of S100a8(pg/ml) | O.D  S100a8 without DTT | O.D  S100a8 with DTT | Concentration of HMGB1(ng/ml) | O.D  HMGB1 without DTT | O.D  HMGB1 with DTT | Concentration of HSP70(pg/ml) | O.D  HSP70 without DTT | O.D  HSP70 with DTT |
| --- | --- | --- | --- | --- | --- | --- | --- | --- | --- | --- | --- | --- | --- | --- |
| .0000 | .0090 | .0100 | .000 | .010 | .008 | .000 | .012 | .011 | .00 | .009 | .012 | .00 | .109 | .110 |
| .3130 | .0700 | .0680 | .130 | .018 | .016 | 31.300 | .054 | .056 | 1.25 | .034 | .032 | 1.56 | .155 | .159 |
| .6250 | .1400 | .1450 | .400 | .038 | .039 | 62.500 | .090 | .093 | 2.50 | .074 | .069 | 3.12 | .238 | .245 |
| 1.2500 | .3000 | .3080 | 1.200 | .089 | .085 | 125.000 | .134 | .130 | 5.00 | .140 | .139 | 6.25 | .388 | .400 |
| 2.5000 | .6250 | .6040 | 3.600 | .220 | .210 | 250.000 | .294 | .312 | 10.00 | .271 | .250 | 12.50 | .603 | .589 |
| 5.0000 | 1.2600 | 1.2100 | 11.000 | .482 | .460 | 500.000 | .602 | .620 | 20.00 | .551 | .535 | 25.00 | 1.101 | 1.112 |
| 10.0000 | 2.4100 | 2.2800 | 33.000 | .954 | .951 | 1000.000 | 1.157 | 1.165 | 40.00 | 1.032 | 1.021 | 50.00 | 1.732 | 1.789 |
|  |  |  | 100.000 | 1.452 | 1.431 | 2000.000 | 2.305 | 2.511 | 80.00 | 2.074 | 1.984 | 100.00 | 2.328 | 2.438 |
